# Analysis and Correction of Dynamic Geometric Misalignment for Nano-Scale Computed Tomography at BSRF

**DOI:** 10.1371/journal.pone.0141682

**Published:** 2015-10-28

**Authors:** Jian Fu, Chen Li, Zhenzhong Liu

**Affiliations:** Research center of digital radiation imaging, Beijing University of Aeronautics and Astronautics, Beijing, People’s Republic of China; University of Notre Dame, UNITED STATES

## Abstract

Due to its high spatial resolution, synchrotron radiation x-ray nano-scale computed tomography (nano-CT) is sensitive to misalignments in scanning geometry, which occurs quite frequently because of mechanical errors in manufacturing and assembly or from thermal expansion during the time-consuming scanning. Misalignments degrade the imaging results by imposing artifacts on the nano-CT slices. In this paper, the geometric misalignment of the synchrotron radiation nano-CT has been analyzed by partial derivatives on the CT reconstruction algorithm and a correction method, based on cross correlation and least-square sinusoidal fitting, has been reported. This work comprises a numerical study of the method and its experimental verification using a dataset measured with the full-field transmission x-ray microscope nano-CT at the beamline 4W1A of the Beijing Synchrotron Radiation Facility. The numerical and experimental results have demonstrated the validity of the proposed approach. It can be applied for dynamic geometric misalignment and needs neither phantom nor additional correction scanning. We expect that this method will simplify the experimental operation of synchrotron radiation nano-CT.

## Introduction

X-ray computed tomography(X-CT) has enabled the non-destructive observation of internal structures and is a powerful analysis tool. It has become popular in medicine, biology and materials science since its introduction in the 1970s [1–11]. Synchrotron radiation CT (SR-CT) is the combination of synchrotron radiation light source and classical X-CT theory [12, 13]. It presents several advantages over conventional x-ray tube sources, including narrow spectrum and high brilliance, and can provide superior imaging contrast and spatial resolution than conventional X-CT, and has therefore attracted significant interests. Over the last few decades, several SR-CT methods have been developed. One of the recent developments is full-field transmission x-ray microscope (TXM) nano-scale X-CT (nano-CT) [14, 15]. With outstanding nano-scale imaging capabilities, it has been applied to a wide range of research work in biological and materials science [15–18]. Many synchrotron radiation facilities have built this kind of experimental system. It has also undergone rapid improvements due to the development of high-efficient x-ray optics.

TXM nano-CT includes essentially two steps: (i) projection data acquisition and (ii) image reconstruction. The first step can be accomplished by combining a sample rotation with TXM. The second step has so far been solved by using analytical and iterative algorithms such as filtered back-projection (FBP) algorithm, algebraic or statistical iteration reconstruction. The coordinate system describing the image reconstruction should coincide with the one describing the projection acquisition geometry. Otherwise geometric misalignment will occur and lead to misalignment of the projection data. If the aforementioned algorithms directly perform reconstruction from the misaligned data, blurring and streaking artifacts will be generated in the CT slice image and cause information loss and false structures [19–26].

The static geometric misalignment in X-CT systems has already been resolved well by many methods. According to the working principle and the implementation procedure, they can be classified in to three groups. The first group requires an additional CT measurement of a correction object as it is described in references [22, 27, 28]. The second one does not place additional markers in the field of measurement [23]. The last group needs neither correction phantom nor additional scanning [20, 21, 24–26, 29–31]. It is based on the reconstructed CT slice images or the recorded original projections. Typically, Viskoe et al adopted a centroid registration algorithm to correct for misalignment in second-generation CT system with an equiangular detector [29]. Rivers et al corrected the horizontal shift of the projection by computing the center of gravity in the sinogram [30]. Donath et al presented a center of mass method to determine the center of rotation [31]. However, for the TXM nano-CT at the Beijing Synchrotron Radiation Facility (BSRF), the scanning time is usually much longer and the geometric misalignment changes over time randomly during the scanning. There is currently a scarcity of studies on dynamic geometric misalignment and its correction. The existing techniques were mainly based on iterative calculations. For example, Donath et al presented three image metrics for the scoring of tomographic reconstructions and an iterative procedure for the determination of the position of the optimum center of rotation. Wang et al developed a LabVIEW-based iterative correction procedure that adjusts the alignment of a gold particle phantom manually by human-computer interaction [19]. They are complicated, time-consuming and not convenient in nano-CT. There remains an important need to develop the correction techniques for dynamic geometric misalignment.

The TXM nano-CT at BSRF operates continuously from 5 keV to 12 keV with fluorescence mapping capability and has a spatial resolution better than 30 nm [15]. In this system, step-shoot scanning is used to obtain the projection data because the x-ray detector takes about two seconds to acquire a single image. Under this scanning, the detector remains stationary and the sample stage rotates discontinuously to sample different view angles. During the rotation, mechanical errors in manufacturing and assembly can cause the jittering of the rotation axis of the sample stage such as runout, wobble and eccentricity. Thermal expansion due to temperature variation and external environmental changes during the time-consuming experiment also have influence on the system. Due to these factors, geometric misalignment always exists and varies over time. Moreover the extremely high precision of nano-CT may reveal mechanical errors that are commonly neglected in conventional tube source X-CT. Therefore, some misalignment correction methods which are effective in tube source X-CT probably become ineffective when applied to nano-CT. Wang et al once developed a LabVIEW-based iterative correction platform for nano-CT that adjusts the alignment manually [19].

In this paper, we reported a geometric misalignment correction method for TXM nano-CT at BSRF and its experimental verification. Firstly, the misalignment was analyzed and decomposed into errors along three axial directions in the scanning coordinate system. The influence of these errors on imaging results was investigated. Then the correction method was described. Finally the numerical simulation and the experimental demonstration using a dataset measured with TXM nano-CT at the beamline 4W1A of BSRF were presented. Compared with the existing techniques, the proposed method can work for random misalignments and moreover needs neither phantom nor additional scanning. It would be helpful to simplify the experimental operation of synchrotron radiation nano-CT and push its future applications.

## Materials and Methods

### Geometric misalignment at nano-CT

**Fig 1:**
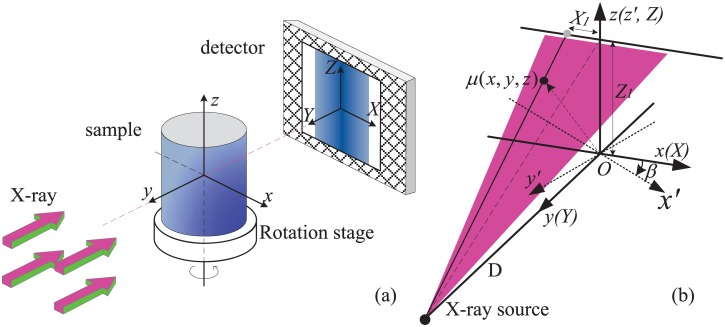
The scanning geometry of nano-CT at BSRF. (a) is the simplified scanning geometry and x-ray optical layout is here ignored. (b) depicts three Cartesian coordinate systems: (X, Y, Z) for projection image acquisition, (*x*, *y*, *z*) for image reconstruction and (*x*′, *y*′, *z*′) for the stage.

Fig 1 is the simplified scanning geometry of nano-CT at BSRF (The x-ray optical layout is here ignored and can be found in [15]). During the scanning, the sample stage rotates step by step for 180°. The x-ray from synchrotron radiation light source hits the sample. The x-ray detector remains stationary and captures the two dimensional projection at each view angle. The three dimensional CT image of the sample can finally be reconstructed by the popular FDK reconstruction algorithm [32]

$$\begin{array}{c} \mu \left(x,y,z\right)=\int_{0}^{2\pi }U^{2}\times P^{\prime }\left(X_{1},Z_{1},\beta \right)d\beta \end{array}$$ (1)

with

$$\begin{array}{c} P^{\prime }\left(X,Z,\beta \right)=\left(P\left(X,Z,\beta \right)\times K\right)*h\left(X\right)/2, \end{array}$$ (2)

$$\begin{array}{c} K=\frac{D}{\sqrt{D^{2}+X^{2}+Z^{2}}}, \end{array}$$ (3)

$$\begin{array}{c} X_{1}=\frac{D(xcos\beta +ysin\beta )}{D+xsin\beta -ycos\beta }, \end{array}$$ (4)

$$\begin{array}{c} Z_{1}=\frac{Dz}{D+xsin\beta -ycos\beta }, \end{array}$$ (5)

and

$$\begin{array}{c} U=\frac{D}{D+xsin\beta -ycos\beta } \end{array}$$ (6)

from the recorded two dimensional projection dataset.

In Eqs (1)–(6), *μ*(*x*, *y*, *z*) represents the linear attenuation coefficient of any reconstructed point in the sample. *P*(*X*, *Z*, *β*) is the acquired projection by the detecting channel (*X*, *Z*) at the view angle *β*. *U* and *K* are the geometric correction factors. *h*(*X*) is the Ram-Lak filter. D is the distance from the rotation center to the x-ray source. Given a reconstructed point (*x*, *y*, *z*), we first calculate the positions of the corresponding detecting channels for each view angle with Eqs (4) and (5). And then the projections recorded by these channels are filtered, summed, averaged and weighted to get the CT image.

For the following discussion on the vertical vibration error of the stage, the parallel-beam imaging geometry in Fig 1a is generalized to the cone-beam geometry in Fig 1b since the parallel-beam reconstruction algorithm does not involve the vertical coordinate (A parallel-beam geometry can be treated as a cone-beam geometry with infinitely small cone-angle.). In Fig 1b, the detector is moved to the position of the rotation center of the sample stage. This movement does not change the validity of this method. Fig 1b depicts three Cartesian coordinate systems: (X, Y, Z) for projection image acquisition, (*x*, *y*, *z*) for image reconstruction and (*x*′, *y*′, *z*′) for the rotation stage. (*x*′, *y*′, *z*′) is the rotation transform version of (*x*, *y*, *z*).

The above FDK algorithm assumes that the coordinate system (*x*′, *y*′, *z*′) keeps stable during the rotation scanning. However, those factors mentioned in the “Introduction” section would cause the instability of the sample stage. This leads to the shift of the coordinate system (*x*′, *y*′, *z*′) randomly and causes geometric misalignment.

### Influence on CT images

Because the shift of the coordinate system (*x*′, *y*′, *z*′) can be treated equivalently as the shift of the coordinate system (*x*, *y*, *z*), the influence of this instability on the image reconstruction can be analyzed by taking the partial derivatives on x, y and z in Eqs (4)–(6). The partial derivative analysis on *y* can be replaced by the one on D since the shift along axis *y* actually changes the value of D.

We first analyze the influence from the shift of D. Eqs (7)–(9) list the partial derivatives on D. In the nano-CT system at BSRF, the value of D (tens of meters) is much bigger than the image size *x* and *z* (from several to tens of micrometers). The x-ray beams are approximately parallel each other and the cone-angle is close to zero. Obviously these partial derivatives in Eqs (7)–(9) approach zero and show that the influence from the shift of D is tiny and can be ignored.

$$\begin{array}{c} \frac{\partial X_{1}}{\partial D}=\frac{\left(xcos\beta +ysin\beta )(xsin\beta -ycos\beta \right)}{{\left(D+xsin\beta -ycos\beta \right)}^{2}}\approx 0, \end{array}$$ (7)

$$\begin{array}{c} \frac{\partial Z_{1}}{\partial D}=\frac{z(xsin\beta -ycos\beta )}{{\left(D+xsin\beta -ycos\beta \right)}^{2}}\approx 0, \end{array}$$ (8)

$$\begin{array}{c} \frac{\partial U}{\partial D}=\frac{xsin\beta -ycos\beta }{{\left(D+xsin\beta -ycos\beta \right)}^{2}}\approx 0. \end{array}$$ (9)

Eqs (10)–(12) and Eqs (13)–(15) list the partial derivatives on *x* and *z*. Obviously the shifts along *x* and *z* will affect the calculation of *X*_1_ and *Z*_1_. The detecting channel recording the projection cannot be accurately determined. So, it is necessary to correct these two shifts in experiments.

$$\begin{array}{c} \frac{\partial X_{1}}{\partial x}\approx \frac{\partial (xcos\beta +ysin\beta )}{\partial x}=cos\beta \end{array}$$ (10)

$$\begin{array}{c} \frac{\partial Z_{1}}{\partial x}\approx \frac{\partial (z)}{\partial x}=0 \end{array}$$ (11)

$$\begin{array}{c} \frac{\partial U}{\partial x}\approx \frac{\partial (1)}{\partial x}=0. \end{array}$$ (12)

$$\begin{array}{c} \frac{\partial X_{1}}{\partial z}\approx \frac{\partial (xcos\beta +ysin\beta )}{\partial z}=0 \end{array}$$ (13)

$$\begin{array}{c} \frac{\partial Z_{1}}{\partial z}\approx \frac{\partial (z)}{\partial z}=1 \end{array}$$ (14)

$$\begin{array}{c} \frac{\partial U}{\partial z}\approx \frac{\partial (1)}{\partial z}=0. \end{array}$$ (15)

Based on the original projection images recorded by the detector, a geometric misalignment correction method was developed. It includes two stages: (i) correction of the vertical shift on axis *z* and (ii) correction of the horizontal shift on axis *x*. The first one is based on the cross correlation of the plane integral curves at each view angle. The second is based on the least-square sinusoidal fitting of the center of mass of the sample. It is the same as the one reported in [30]. The shift correction on *z* is executed first because it is independent of the shift along *x*.

### Correction of the vertical shift

**Fig 2:**
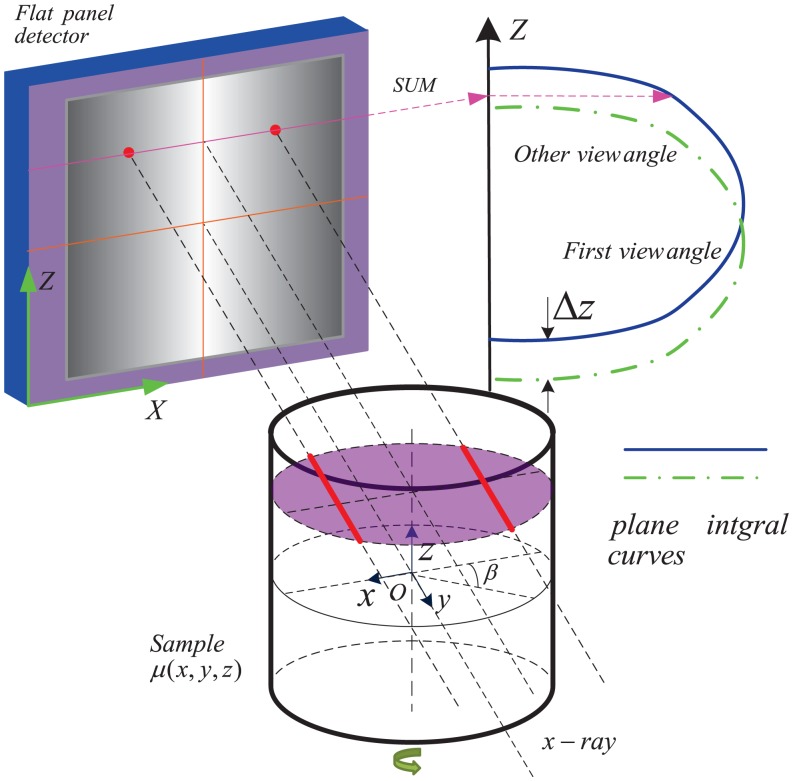
The plane integral curves of the sample with the shift along vertical axis *z*.

This correction involves the concept of the plane integral curve of the sample, depicted in Fig 2. In CT, each detecting channel provides the line integral of the sample and the sum of the line integrals along the row of the detector produces the plane integral of the cross-section. The plane integral curve is formed if this sum operation is executed along all the rows of the detector. For parallel-beam imaging geometry in Fig 1, there exists a fact that the plane integral curve does not vary over view angle if the sample has no change during the scanning. So the vertical shift of the stage just leads to the vertical shift of this curve and does not change its shape and size. When the stage has a vertical shift Δ*z*, this curve also shifts vertically Δ*z*. The idea of this correction is to measure this shift Δ*z* for every view angle and input it into Eq (5) to correct the projection address.

The vertical shift Δ*z* can be determined by the concept of the cross correlation. In time-domain signal processing, the cross correlation is a measure of similarity of two signals as a function of the lag of one relative to the other. In our case, it is adopted to find the position at which two space-domain signals have the biggest similarity. Obviously it reaches a peak when two plane integral curves coincide. So the position of the peak of the cross correlation curve provides the vertical shift Δ*z*. The cross correlation discrete formula is expressed by Eq (16). Here *P*_*p*_(*Z*, *β*_1_) and *P*_*p*_(*Z*, *β*_*i*_) represent two plane integral curves at the first view angles *β*_1_ and the *ith* view angle *β*_*i*_. N is the number of the data points in the plane integral curve. *m* represents the distance between two signals. This operation is executed in space-domain. It can also be implemented by calculating the cross power spectrum in frequency-domain. The results are the same.

$$\begin{array}{c} R_{P_{p}\left(Z,\beta_{1}\right)P_{p}\left(Z,\beta_{i}\right)}\left(m\right)=\frac{1}{N}\sum_{Z=0}^{N}P_{p}\left(Z,\beta_{1}\right)P_{p}\left(Z+m,\beta_{i}\right). \end{array}$$ (16)

The implementation procedure for this correction is therefore as follows: (i) Perform a logarithm operation on the recorded image sequence to get *P*(*X*, *Z*, *β*); (ii) Sum *P*(*X*, *Z*, *β*) along *X* to get *P*_*p*_(*Z*, *β*); (iii) Do the operation in Eq (16) for all view angles and find the positions corresponding to the peaks; (iv) Correct the vertical shift by inputting the correction value to Eq (5).

### Correction of the horizontal shift

**Fig 3:**
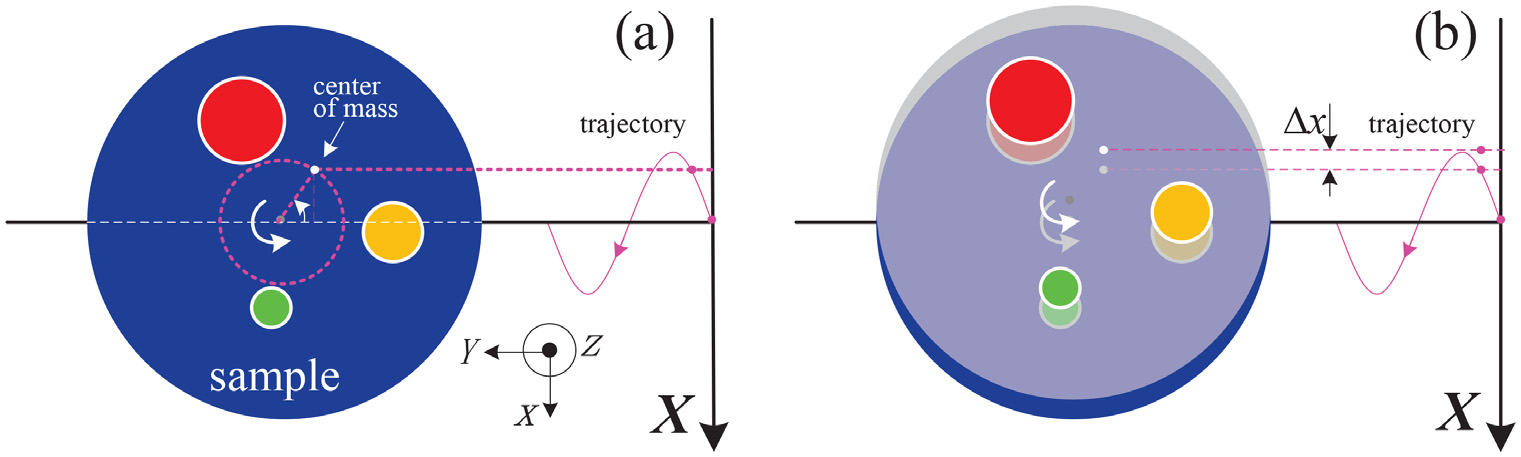
The trajectory of the center of mass of the sample. (a) depicted the rotation trajectory of the center of mass of the sample in the two dimensional plane without the horizontal shift along *x*. (b) shows the position shift of the mass of center when the horizontal shift along *x* happens.

For parallel-beam imaging geometry in Fig 1, the correction of the horizontal shift can be simplified to the two dimensional plane (*x*, *y*) when the above correction of the vertical shift is completed. This correction of the horizontal shift is based on a fact that the rotation trajectory of the center of mass of the sample should be a sinusoidal curve in the two dimensional plane, depicted in Fig 3. When the horizontal shift along *x* happens, the actual rotation trajectory of the center of mass will be a curve vibrating around the ideal sinusoidal curve. So the horizontal shift can be corrected if the ideal sinusoidal curve is determined from the actual trajectory.

In practice, the ideal sinusoidal curve can be estimated from the actual one using the least-square sinusoidal curve fitting. This operation can be implemented by adopting the Matlab function “lsqcurvefit” or other tools. The actual curve is formed by calculating the coordinate *X* of the center of mass of the sample for each view angle using Eq (17) at the selected *Zth* cross-section. Here M is the number of the detecting channels along the row direction.

$$\begin{array}{c} C_{mX}\left(\beta \right)=\frac{1}{\sum_{i=0}^{M}P\left(X_{i},Z,\beta \right)}\sum_{i=0}^{M}P\left(X_{i},Z,\beta \right)X_{i} \end{array}$$ (17)

The implementation procedure of this correction is listed as follows: (i) Perform a logarithm operation on the recorded image series to get *P*(*X*, *Z*, *β*); (ii) Select one cross-section to calculate the center of mass by fixing the value of *Z*; (iii) Do the operation in Eq (17) for all view angles and get the actual rotation trajectory of the center of mass; (iv) Do least-square sinusoidal curve fitting to estimate the ideal trajectory from the actual one in step (iii) by using the Matlab function “lsqcurvefit” or other tools; (v) Correct the horizontal shift by inputting the difference between the actual trajectory and the fitted one into Eq (4).

As stated by Donath et al [31], this kind of correction approaches based on the center of mass is sensitive to noise. In our experiment, we averaged the sinograms of three adjacent layers to suppress the influence from noise. One can also do this correction for many layers and average the results to let it more robust to noise.

## Numerical simulation

Numerical simulations were performed to validate the proposed method. The phantom is the well-known Shepp-Logan phantom with a size 7.5 × 7.5 × 7.5 *μm* ^3^ and consists of some ellipsoids with different diameters, which are attributed different linear attenuation coefficients ranging from 0 to 1. The detector has 256 × 256 pixels with a size 30 nm for each pixel. The simplified imaging geometry in Fig 1 was used to acquire the two dimensional projection images. They were calculated using the analytical forward projection method which is based on the general equations of ellipsoids and lines in space. The random ranges of shifts along *x*, *y* and *z* are set to be [−150*nm*, 150*nm*], [−150*mm*, 150*mm*] and [−150*nm*, 150*nm*] respectively.

**Fig 4:**
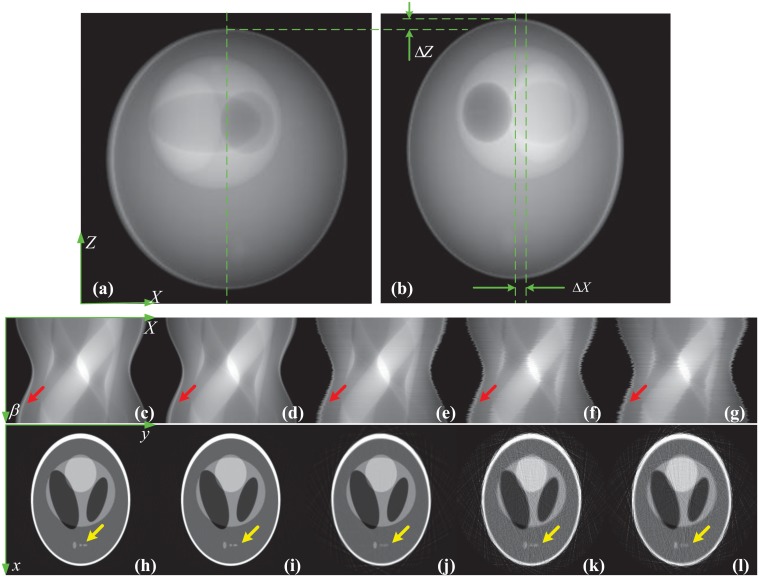
The influence of the geometry misalignment on the CT images. (a) and (b) are two projections under two different view angles. (c)-(g) are the projection sinograms and (h)-(l) the reconstructed CT images. (c) and (h) correspond to the case without jittering, (d) and (i) with shift along axis *x*, (e) and (j) with shift along axis *y*, (f) and (k) with shift along axis *z* and (g) and (l) with shifts along *x*, *y* and *z* simultaneously.

Fig 4 shows the influence of the geometric misalignment by presenting the imaging results of the 160th row in the detector. Fig 4a and 4b are two projections under two different view angles. They shows clearly the horizontal and vertical shifts of the stage. Fig 4c–4g are the projection sinograms and (h)-(l) the reconstructed CT images. Fig 4c and 4h correspond to the case without shift, (d) and (i) with shift along axis *y*, (e) and (j) with shift along *x*, (f) and (k) with shift along *z* and (g) and (l) with shifts along *x*, *y* and *z* simultaneously. The red arrows in Fig 4e, 4f and 4g mark the sawtooth-shaped edges caused by these shifts of the stage. The regions of interest marked by the yellow arrows in Fig 4j, 4k and 4l show that the shifts along *x* and *z* blur the CT image and make it difficult to recognize some structure details. In contrast, the edges in Fig 4c and 4d are continuous and the structures in Fig 4h and 4i are clear. Fig 4 supports the above analysis based on the partial derivatives that the shifts along axis *x* and *z* must be calibrated in experiments and the shift along axis *y* can be ignored.

**Fig 5:**
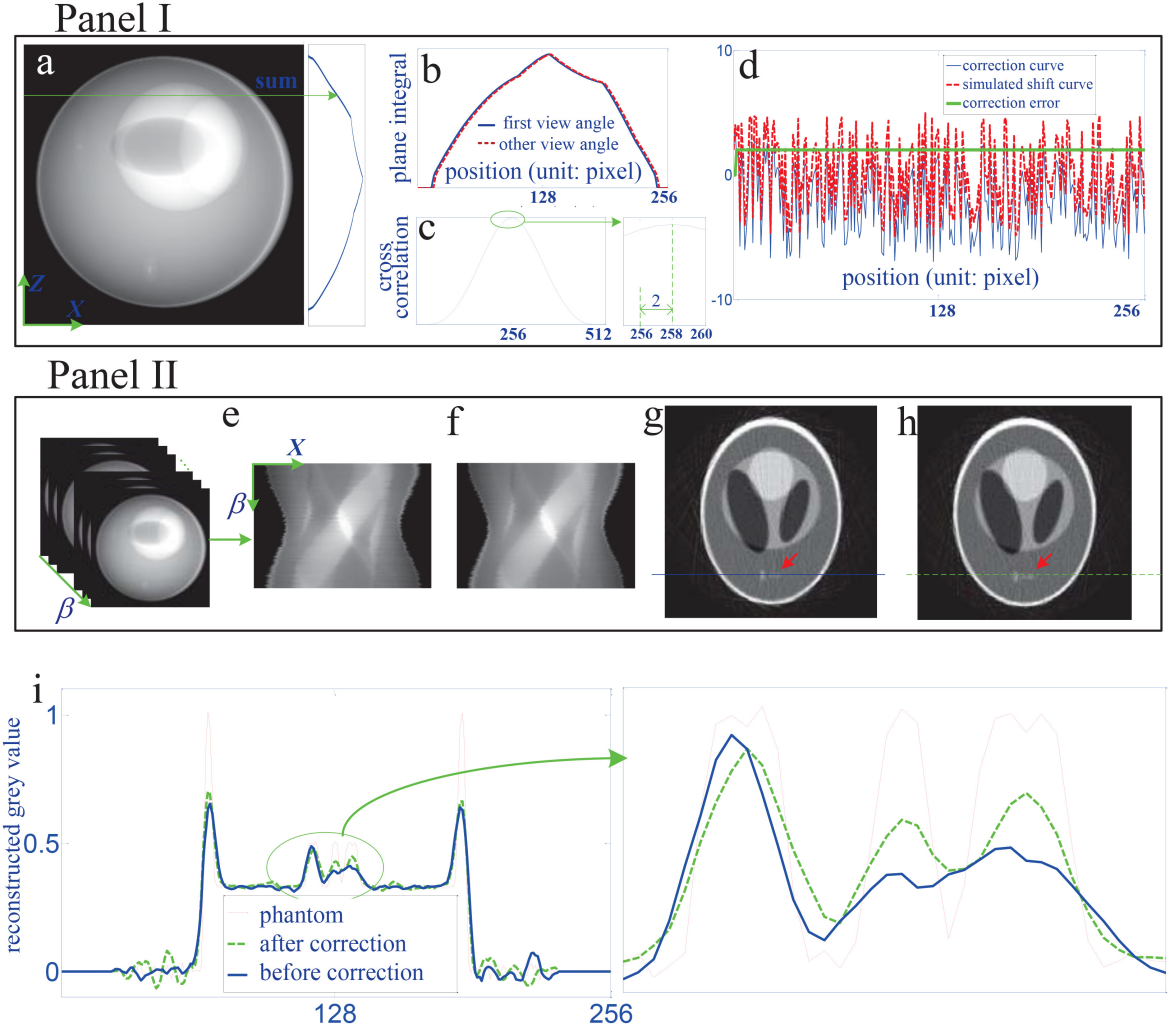
The correction procedure and results for the vertical shift. (a) shows how to produce the plane integral curve at the first view angle. (b) depicts the vertical shift between two view angles by the plane integral curves. (c) is the cross correlation curve of the two signals in (b). (d) compares the calibrated curve with the simulated shift curve. (e) shows how to form the sinogram from the recorded two dimensional projection image sequence. (f) is the calibrated sinogram. (g) and (h) are the reconstructed CT images by the algorithm in Eq (1) with the sinogrms in (e) and (f) respectively. (i) provides the grey value profiles of the 200th row of the images in (g), (h) and the phantom.

Fig 5 shows the correction procedure and results for the vertical shift along *z*. Panel I in Fig 5 is the step for producing the correction curve. In panel I, the plane integral curve at the first view angle was calculated by the sum operation along the horizontal direction of the recorded projection image and displayed in Fig 5a. Using the same operation, this curve can be drawn for every view angle. Fig 5b presents the plane integral curve at the first view angle marked by the solid blue line, and the one at some other view angle marked by the dashed red line. Obviously, there exists an interval between these two curves due to the vertical shift. Fig 5c shows the cross correlation result of these two curves. We can find that the peak of the cross correlation appears at the position indexed by 258. So the correction value for this view angle is 2 since the center of the position index of the plane integral curve is 256. Doing the operations in Fig 5c for all view angles leads to the correction curve in Fig 5d. In this figure, the dashed red line is the simulated vertical shift curve, the solid blue one the correction curve and the bold green one the correction error. We would like to make two remarks on Fig 5d. First, it demonstrates that the proposed method can correct the vertical shift. Second, it shows that the correction error is a constant and always equals 2. It is caused by the vertical shift at the first view angle. In our method, we selected the plane integral curve at the first view angle as the standard one and made the cross correlation calculation between it and the curves at other view angle. So the correction error corresponds to the vertical shift at the first view angle. This error does not affect the image reconstruction since every cross-section can be treated as the central plane in the parallel beam imaging geometry.

Panel II in Fig 5 demonstrates the correction effect by displaying the imaging results of the 160th row in the detector. Fig 5e and 5f are the sinograms before and after the correction. Fig 5g and 5h are the corresponding CT slice images. Observing the regions of interest marked by the red arrows in Fig 5g and 5h, we can find that the result after correction is much closer to the phantom than the one before correction. It validates the proposed correction method for the vertical shift. Fig 5i compares the grey value profiles of the 200th row of the images with the phantom and supports quantitatively this conclusion.

**Fig 6:**
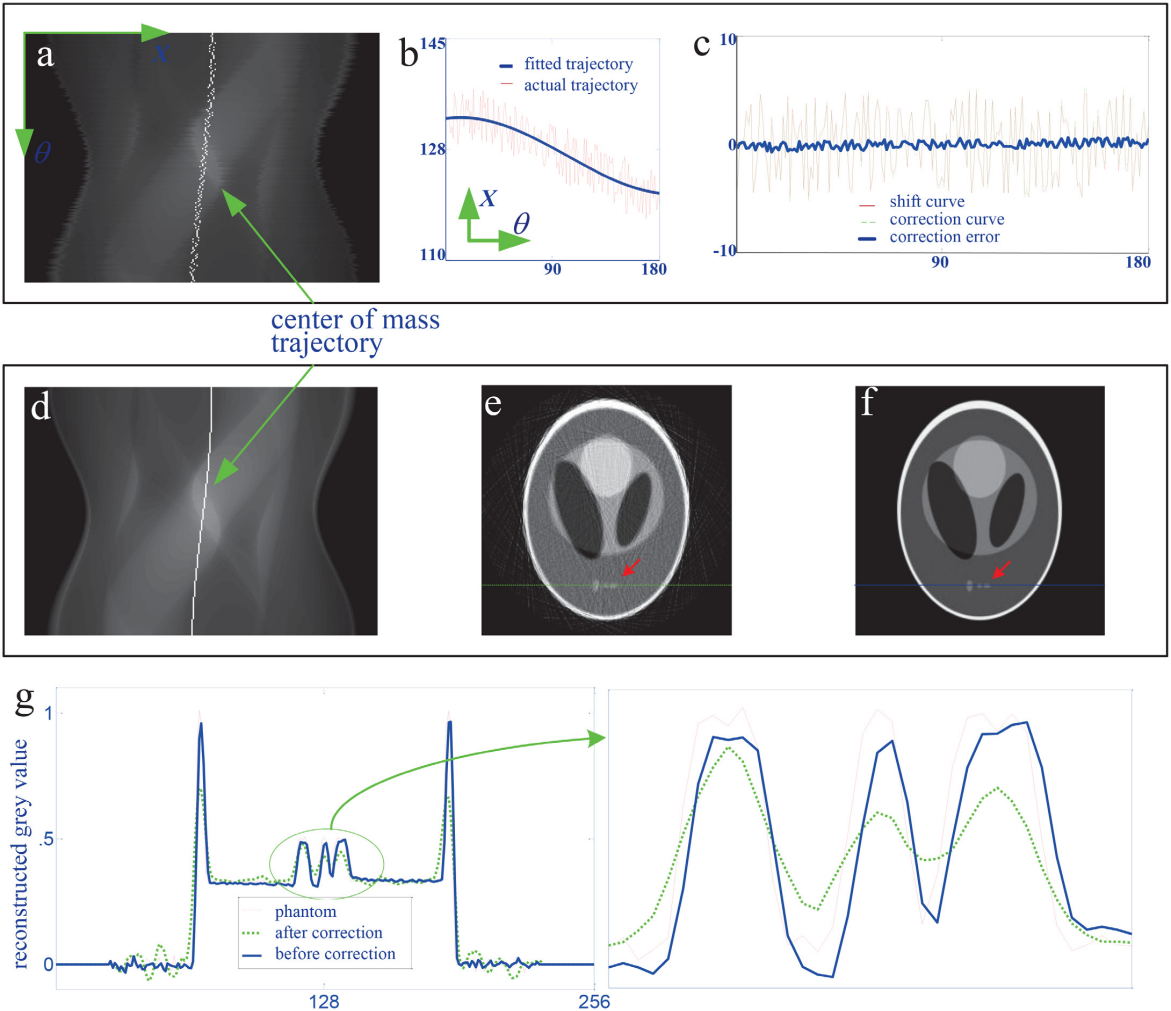
The correction procedure and results for the horizontal shift. (a) shows the actual trajectory of the center of mass of the phantom. (b) presents the fitted trajectory using Matlab function “lsqcurvefit”. (c) compares the calibrated curve with the simulated shift curve. (d) is the corrected trajectory of the center of mass of the phantom. (e) and (f) are the reconstructed CT images by the algorithm in Eq (1) with the sinogrms in (a) and (d) respectively. (i) provides the grey value profiles of the 200th row of the images in (e), (f) and the phantom.

Fig 6 shows the correction procedure and results for the horizontal shift along *x*. Panel I in Fig 6 is the step for producing the correction curve. In panel I, the actual trajectory of the center of mass of the phantom was first calculated using Eq (17) for the sinogram in Fig 5f and represented by the discontinuous white curve in Fig 6a. Fig 6b displays the fitted trajectory with the Matlab function “lsqcurvefit”. The shift curve, the correction curve and their difference are drawn in Fig 6c. The correction curve clearly approaches the shift curve and the error is smaller than 0.5 pixel. The error is caused by the numerical calculation and the discretization of the image.

Panel II in Fig 6 demonstrates the correction effect by displaying the imaging results of the 160th row in the detector. Fig 6d shows the calibrated trajectory of the center of mass of the phantom. Compared with the one before correction in Fig 6a, it is much more continuous. Fig 6e and 6f are the CT slice images reconstructed by Eq (1) with the sinograms in Fig 6a and 6d. Observing the regions of interest marked the red arrows in Fig 6e and 6f, we can find that the result after correction matches the phantom much better than the one before correction. It validates the proposed correction method for the horizontal shift. Fig 6g compares the grey value profiles of the 200th row of the images with the phantom and supports quantitatively this conclusion.

## Experiments

The experimental dataset that was measured to test the proposed correction method was recorded with the TXM nano-CT setup at the beamline 4W1A of BSRF. The sample was a ZrB_2_/SiC nanocomposite ceramic. It was fabricated by mixing nanosized SiC particle (100 nm) into microsized ZrB_2_-based (2 *μ*m) nanocomposite ceramics with spark plasma sintering (SPS). The sample was first crushed, ground and sieved to 250 mesh to achieve particles with a size 60 *μ*m. AB epoxy adhesive was used to fix the sample particle on the top of a pin under the help of an optical microscope. Finally the pin with the particle was mounted to the rotation sample stage. Some gold particles with size smaller than 3 *μ*m were also adhered to the sample as image quality indicators.

**Fig 7:**
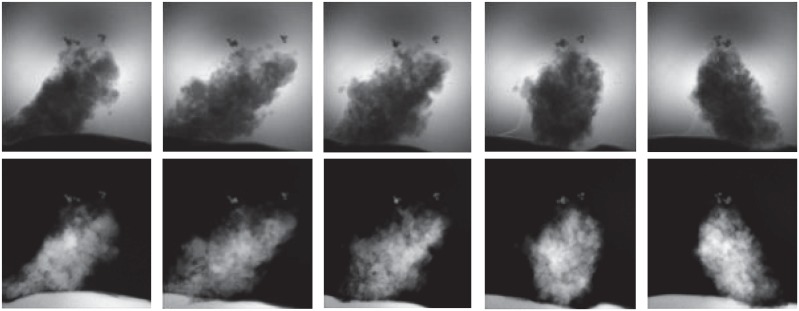
The TXM nano-CT projection images of ZrB_2_/SiC nanocomposite ceramic. The first row is the original images recorded by CCD camera under five view angles. The second row is the ones after logarithm operation.

The general description of the TXM nano-CT setup at the beamline 4W1A of BSRF can be found in [15]. It is primarily composed of a condenser, sample stage, zone plate and CCD detector. The SR x-ray beam is focused onto the sample by a elliptically shaped capillary condenser. Then the objective zone plate produces a magnified projection image of the sample on a scintillator crystal. The resulting visual image is then further enlarged with a microscope objective lens and captured by a 16-bit 1024 × 1024 CCD camera. When the size of the sample is smaller the depth of focus of the microscope, this imaging layout can be equivalently treated as the parallel-beam imaging geometry in Fig 1a. The x-ray energy was set to be 8keV. 360 projections was acquired over 180° rotation with 20 seconds exposure per projection. Fig 7 shows some TXM nano-CT projection images of ZrB_2_/SiC nanocomposite ceramic under different view angles. The first row is the original images recorded by CCD camera and the second row is after processing logarithm operation.

**Fig 8:**
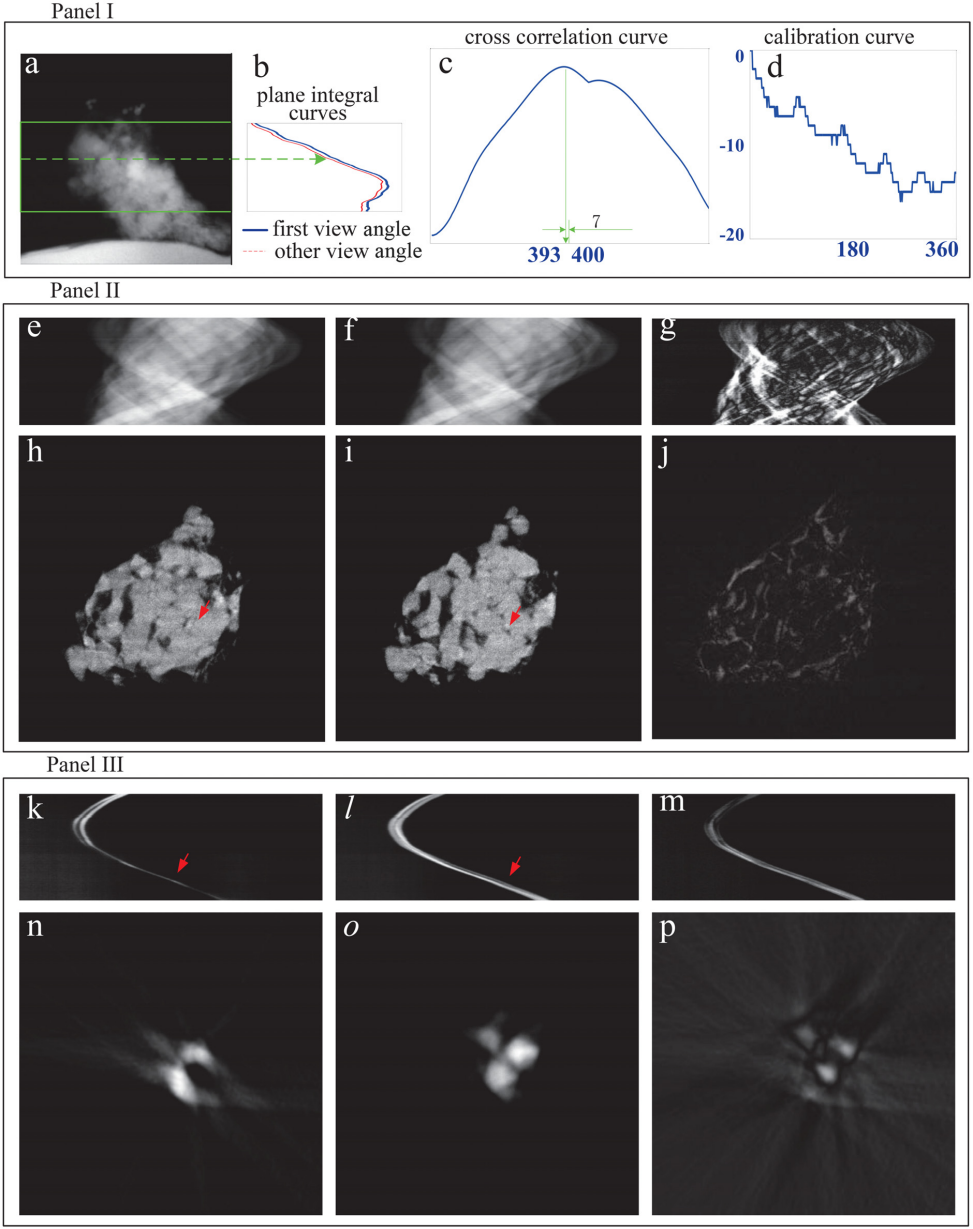
The correction procedure and results for the vertical shift with the TXM nano-CT experimental data. (a) is one of the two dimensional projection images after logarithm operation. (b) depicts the plane integral curve at the first view angle marked by the solid blue line and the one at other view angle marked by the dashed red line. (c) shows the cross correlation result of these two curves in (b). (d) is the correction curve. (e) and (h) are the sinogram and the CT image of the 690th row of the projection before correction. (f) and (i) are after correction. (g) shows the difference between (e) and (f). (j) shows the difference between (h) and (i). (k) and (n) are the sinogram and the CT image of gold particles before correction. (l) and (o) are after correction. (m) shows the difference between (k) and (l). (p) shows the difference between (n) and (o).

Fig 8 presents the correction procedure and results for the vertical shift with the experimental data. Panel I in Fig 8 shows how to calculate the correction curve. In order to avoid the influence from the non-uniformity of the irradiation field (see the first row in Fig 7), we selected the region marked by the green rectangle with a size 400 × 1024 in Fig 8a to calculate the plane integral curve. In panel I, Fig 8a is one of the two dimensional projection images after logarithm operation. Fig 8b displays the plane integral curve at the first view angle marked by the solid blue line and the one at other view angle marked by the dashed red line. Fig 8c shows the cross correlation result of these two curves in Fig 8b. The peak of the cross correlation appears at the position indexed by 393. So the correction value for this view angle is 7 since the center of the position index of the plane integral curve is 400. The correction curve in Fig 8d is depicted after doing the operations in Fig 8c for all 360 view angles. Obviously, this curve shows that the stage is descending vertically with respect to view angle during the scanning. Although it exhibits some periodicity, this shift is generally random.

Panel II in Fig 8 shows the correction results of one typical slice, the 690th row in the detector. Fig 8e and 8h are the sinogram and the CT image before correction. Fig 8f and 8i are after correction. Fig 8g and 8j show the differences of sinograms and CT images before and after correction. Some observations can be made for Panel II. Due to the vertical shift of the stage, the sinogram of the 690th row is incomplete and misaligned by the neighbor rows. Before correction, some structures marked by the red arrow are distorted or disappear and the edge is blurred. In contrast, these problems are mitigated after correction. The results in Panel II demonstrate the validity of the proposed correction method for vertical shift of the stage.

Panel III in Fig 8 repeats the demonstration on the correction performance with the experimental data of gold particles adhered to the sample. Fig 8k and 8n are the sinogram and the CT image before correction. Fig 8l and 8o are after correction. Fig 8m and 8p shows the differences of sinograms and CT images before and after correction. The observation to Panel III provides the same conclusion as the one to Panel II.

**Fig 9:**
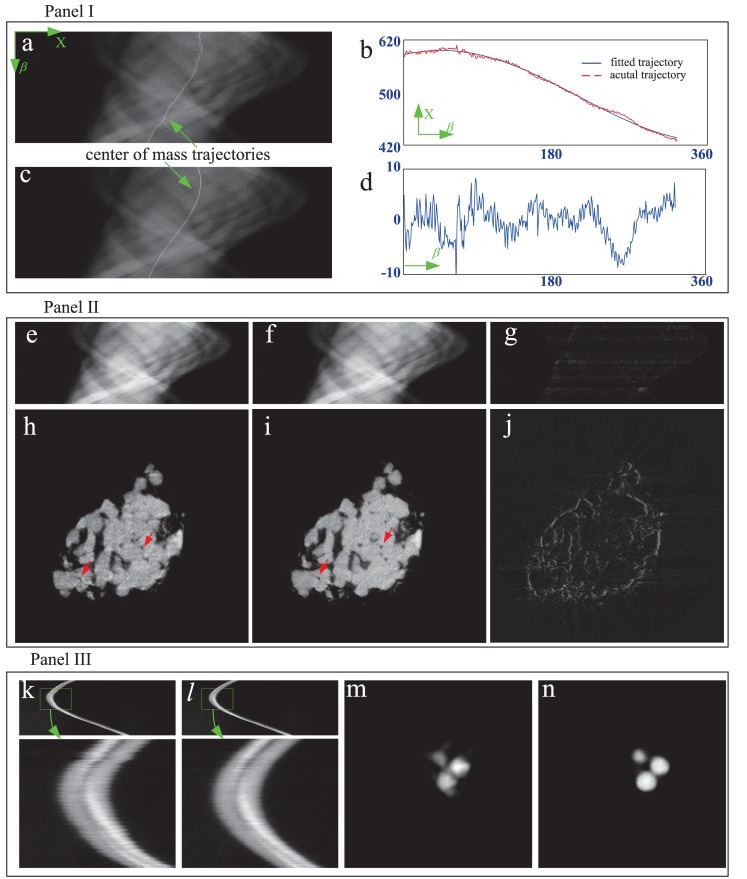
The correction procedure and results for the horizontal shift with the TXM nano-CT experimental data. (a) is the sinogram of the 690th row of the projection with a size 360 × 1024 after the vertical shift correction. (b) depicts the actual trajectory of the center of mass and the fitted one. (c) is the calibrated sinogram and the trajectory of the center of mass using the correction curve in (d). (e) and (h) are the sinogram and the CT image before correction. (f) and (i) are after correction. (g) and (j) show the differences of sinograms and CT images before and after correction. (k) and (m) are the sinogram and the CT image of gold particles before correction. (l) and (n) are after correction.

Fig 9 presents the correction procedure and results for the horizontal shift with experimental data. Panel I in Fig 9 shows how to calculate the correction curve. Fig 9a is the sinogram of the 690th row with a size 360 × 1024 after the vertical shift correction. It also displays the actual trajectory of the center of mass of the sinogram calculated by Eq (17). Fig 9b depicts the actual trajectory of the center of mass and the fitted one. The adopted fit function in Matlab is *y* = *Asin*(*x*/360*π+*B*)+*C*. In this function, *y* represents the trajectory index and *x* the view angle index. The fitted results are *A* = 87.0621, *B* = 1.1457 and *C* = 512.8613. Fig 9c shows the sinogram and the trajectory of the center of mass calibrated using the curve in Fig 9d.

Panel II in Fig 9 shows the correction results of the 690th row. Fig 9e and 9h are the sinogram and the CT image before correction. Fig 9f and 9i are after correction. Fig 9g and 9j show the differences of sinograms and CT images before and after correction. Obviously, the structure distortion and the edge blur marked by the red arrows disappear after correction. The results in Panel II demonstrate the validity of the proposed correction method for horizontal shift of the stage.

Panel III in Fig 9 repeats the demonstration on the correction performance with the experimental data of gold particles adhered to the sample. Fig 9k and 9m are the sinogram and the CT image before correction. Fig 9l and 9n are after correction. Obviously the observation to Panel III provides the same conclusion as Panel II.

**Fig 10:**
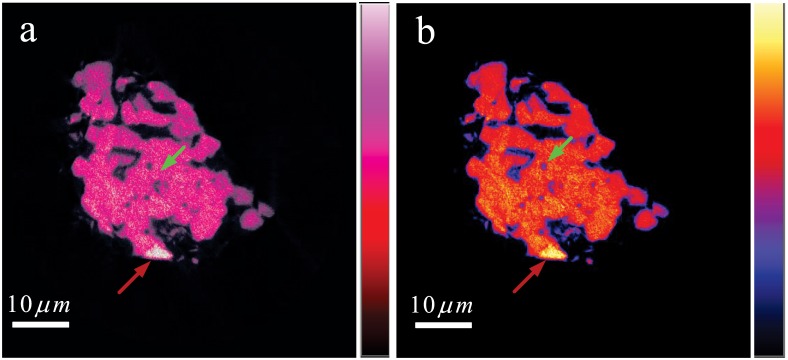
The false color map of the typical slice in Fig 9i.

Fig 10 is the false color map of the typical slice in Fig 9i. It clearly provides the distribution of the nanosized SiC particles in microsized ZrB_2_-based nanocomposite ceramics, marked by the green arrow in Fig 10a. The imaging results show the existence of aggregation of SiC particles marked by the red arrows in Fig 10a and 10b. It also allows for the inspection of holes in the sample marked by the green arrow in Fig 10b. This information is helpful to the performance analysis and improvement of this nanocomposite. A comparison between Figs 8h and 10 demonstrates the validity of the proposed correction method.

## Discussion and Conclusion

In summary, we have analyzed the geometric misalignment in the TXM nano-CT at BSRF and established a correction method for this system. We have also demonstrated its validity and performance both numerically with simulation and experimentally with real data. This method is based on the cross correlation of the plane integral curves and the least-square sinusoidal fitting of the center of mass of the projection sinogram. It avoids the use of a correction phantom and additional scanning and can work for dynamic geometric misalignment. Additionally it can be implemented automatically without human supervision and will simplify the experimental operation of the TXM nano-CT.

It should be pointed that the proposed method is applicable only for rigid body motion and only applies for translation correction, which is fine for most of the cases in nano-CT where samples are not too radiation sensitive and do not deform during the scans. Additionally it assumes that the motions introduced by the wobble of the rotary stages are negligible vertically and can be approximated as simple horizontal displacement. The technique for vertical alignment does not generate cumulative errors, which is an asset in regard to other approaches registering consecutive projections. In the future, other fitting approaches could be used to further improve the correction accuracy.
